# A novel strategy to uncover specific GO terms/phosphorylation pathways in phosphoproteomic data in *Arabidopsis thaliana*

**DOI:** 10.1186/s12870-021-03377-9

**Published:** 2021-12-14

**Authors:** Denise S. Arico, Paula Beati, Diego L. Wengier, Maria Agustina Mazzella

**Affiliations:** 1 INGEBI-CONICET Instituto de Investigaciones en Ingeniería Genética y Biología Molecular “Dr. Héctor Torres”, Vuelta de Obligado 2490, 1428 CABA, Argentina; 2 Department of Chemical Engineering, Stanford University, 443 Via Ortega, Stanford, CA 94305 USA

**Keywords:** Phosphoproteome, Gene ontology, *Arabidopsis thaliana*, Reference datasets, Etiolation

## Abstract

**Background:**

Proteins are the workforce of the cell and their phosphorylation status tailors specific responses efficiently. One of the main challenges of phosphoproteomic approaches is to deconvolute biological processes that specifically respond to an experimental query from a list of phosphoproteins. Comparison of the frequency distribution of GO (Gene Ontology) terms in a given phosphoproteome set with that observed in the genome reference set (GenRS) is the most widely used tool to infer biological significance. Yet, this comparison assumes that GO term distribution between the phosphoproteome and the genome are identical. However, this hypothesis has not been tested due to the lack of a comprehensive phosphoproteome database.

**Results:**

In this study, we test this hypothesis by constructing three phosphoproteome databases in *Arabidopsis thaliana:* one based in experimental data (ExpRS), another based in in silico phosphorylation protein prediction (PredRS) and a third that is the union of both (UnRS). Our results show that the three phosphoproteome reference sets show default enrichment of several GO terms compared to GenRS, indicating that GO term distribution in the phosphoproteomes does not match that of the genome. Moreover, these differences overshadow the identification of GO terms that are specifically enriched in a particular condition. To overcome this limitation, we present an additional comparison of the sample of interest with UnRS to uncover GO terms specifically enriched in a particular phosphoproteome experiment. Using this strategy, we found that mRNA splicing and cytoplasmic microtubule compounds are important processes specifically enriched in the phosphoproteome of dark-grown Arabidopsis seedlings.

**Conclusions:**

This study provides a novel strategy to uncover GO specific terms in phosphoproteome data of Arabidopsis that could be applied to any other organism. We also highlight the importance of specific phosphorylation pathways that take place during dark-grown Arabidopsis development.

**Supplementary Information:**

The online version contains supplementary material available at 10.1186/s12870-021-03377-9.

## Background

While genes are the most fundamental heritable biological units, proteins are the functional actors of most biological processes. At that level, changes in post-translational modifications (PTM) enable the fast modulation of protein function in response to endogenous or environmental cues, a requirement to the crucial changes in gene expression that ensures cell adaptation. For example, some typical targets of PTM are transcription factors that may become activated or inactivated by phosphorylation, and their regulation modifies the genetic program of the plant cell [1–4]. Also, many kinases are subjected to phosphorylation, transitioning from inactive to active phosphotransferases, implying a self-propagating phosphorylation cascade [5]. Protein stability and subcellular localization, such as nuclear translocation can also be regulated by phosphorylation [6, 7]. In Arabidopsis, the role of protein phosphorylation in regulating cellular processes is supported by the identification of numerous protein kinases and phosphatases in the genome [8]. Because of the stimuli-dependent nature of protein phosphorylation, a comprehensive mapping of the Arabidopsis phosphoproteome has been technically challenging.

Today, we count with an expression atlas combining RNA-sequencing of transcriptome and mass spectrometry of proteome and phosphoproteome from 30 tissues of Arabidopsis [9]. Biological complexity and diversity involve that one genome gives rise to multiple transcriptomes, multiple proteomes and multiple phosphoproteomes in a context-dependent manner. A careful and exhaustive analysis by Mergner and collaborators showed that among the 30 tissues analyzed in the Arabidopsis atlas, 25,158 transcripts out of the 27,655 protein-coding genes (90%) were identified; 18,210 (66%) were expressed as proteins, and 8577 (47%) of them were phosphorylated in at least one instance [9]. Every tissue in Arabidopsis atlas exhibits on average ~ 17,600 transcripts, ~ 14,430 proteins and ~ 14,689 phosphorylation sites [9]. Interestingly, approximately half of every proteome in that study is composed of the same core proteins, and the other half includes tissue-specific or enriched proteins [9]. So, the relationship between genome-transcriptome-proteome-phosphoproteomes is complex, challenging our ability to perform meta-analysis to compare and determine overall or specific trends.

One of the most frequent biological data-mining practices is the GO (Gene Ontology) enrichment analysis on gene sets [10]. GO term analysis is used to identify biological processes (BP), molecular functions (MF) or cellular components (CC) that are particularly over or under represented in a list of genes or proteins, given a certain condition. This analysis compares the distribution of GO terms in a sample set of interest versus that observed in the reference set (genome). So, if a GO term is more or less frequent in the sample set than in the reference set, this indicates functional specificity. Integrative analysis of phosphoproteomes is difficult because of its incomplete coverage and technical-specific bias generated in experimental workflows [11]. For example, the most used buffer during phosphoprotein sample preparation unexpectedly activates signalling events [12]. Also, if we are studying phosphoproteome and signal transduction, it is expected to find an enrichment of kinases and phosphatases [13]. Furthermore, some proteins will never be phosphorylated. So, the direct comparison of a phosphoproteome sample set with the genome reference sets, that implicitly assumes equal GO term distributions between the whole phosphoproteome and the genome, might not be correct. This also suggests that we are missing fundamental data for this comparison: a comprehensive phosphoproteome. This can be achieved with empirical data, but it’s a long and expensive work in process, bound to be biased by the unbalanced experimental conditions tested and techniques employed. Alternatively to empirical data, phosphorylation prediction can be used to construct high confidence, predicted phosphoproteomes, but bearing in mind that a complete predicted phosphoproteome has the risk of having some bias based on the algorithms used and the selection of prediction parameters.

To test the hypothesis that the GO term distribution in the phosphoproteome is similar to that in the genome, we built three phosphoproteomics databases of reference from *Arabidopsis thaliana* to compare against the genome. Our results show that GO term distributions for the phosphoproteomes of reference are different from the genome in the three ontologies (BP, MF and CC), revealing that enriched GO terms in a phosphoproteome sample would be systemically enriched in phosphorylation experiments independently of the phosphoproteome sample background, overshading those GO terms specific to a sample of interest. We also show a strategy to uncover GO terms specifically enriched in a particular phosphoproteomic experiment from those core GO terms enriched in any phosphoproteome. Moreover, we tested our strategy by performing GO enrichment analysis comparing in-house unpublished dark-grown (etiolated) Arabidopsis seedlings phosphoproteome to identify GO terms enriched as a direct consequence of skotomorphogenesis.

## Results

### Construction of reference databases of phosphoproteomic data

**Fig. 1:**
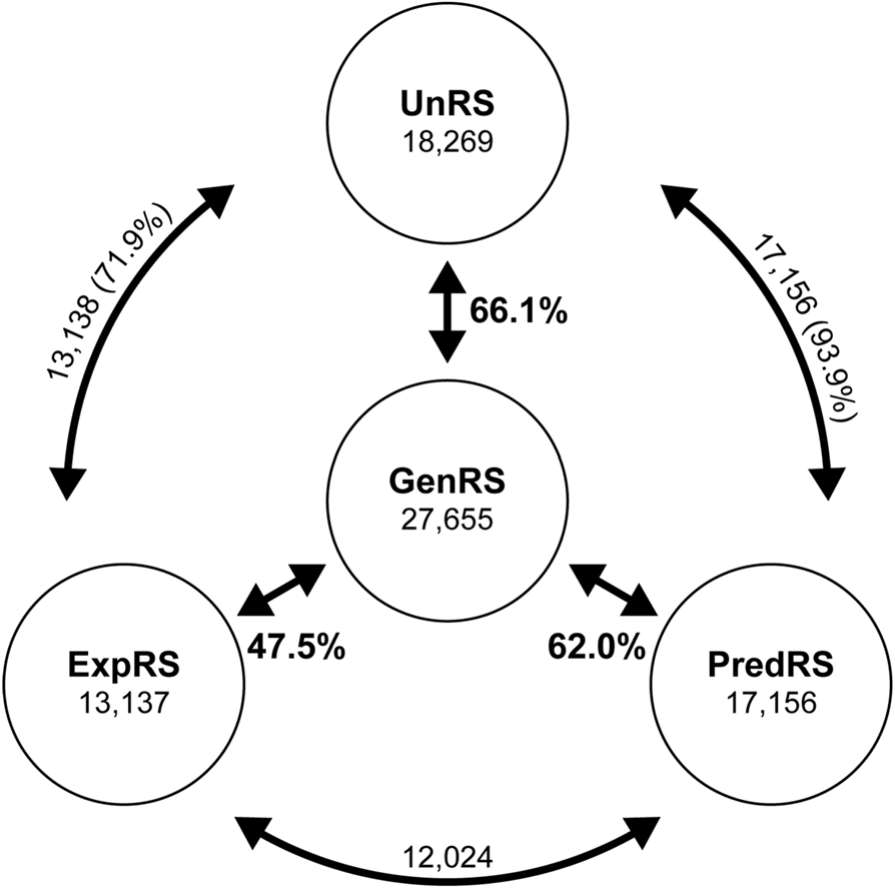
Scheme representing the relations among the three phosphoproteome reference sets ExpRS, PredRS, UnRS and the GenRS. The number of genes for each dataset is indicated below the name. The number of genes shared between pairs of phosphoproteome datasets is indicated beside each arrow. Percentages among relations are indicated

We constructed three phosphoproteome datasets based on data and algorithms available in the public domain. An experimental reference set (ExpRS) was built with phosphoproteins identified and published in 55 available Arabidopsis phosphoproteome experiments (see Methods). A predicted reference set (PredRS) was built with Arabidopsis proteins predicted to be phosphorylated in serine, threonine and tyrosine. To do this, we used two representative computational tools, MusiteDeep and PhosPhAt, that have been shown to have a good performance with high specificity and sensitivity [14] (see Methods). Figure 1 shows the relation between the reference sets. The last reference database was derived from Araport11, the complete re-annotation of the *Arabidopsis thaliana* GenRS, and contains 27,655 protein-coding genes [15]. In summary, ExpRS includes 13,137 genes (47.5% of GenRS) while PredRS includes 17,156 genes (62.0% of GenRS). ExpRS and PredRS share 12,024 genes (Fig. 1). A total of 5132 genes in the PredRS were not present in the ExpRS indicating that the proteins that encode those genes might have not yet been identified experimentally. Interestingly, we found 1113 phosphoproteins in ExpRS that could not be predicted unless the parameters for each predictor were relaxed to the default values (notice that when default values were applied almost the total genome is predicted to undergo phosphorylation, see Methods). This suggests that although these proteins are phosphorylated, they do not fulfill the strict parameters that we used to build the PredRS. So, the use of high stringency for phosphorylation prediction represents a low cost in terms of experimental phosphoproteins that could not be predicted (8.5% 1113 out of 13,137). These proteins represent the false negatives lost when parameters are set to prevent false positives. So, as a complete intersection of ExpRS and PredRS is not achieved (see Methods) we constructed a third dataset, union reference set (UnRS), that corresponds to the fusion of ExpRS and PredRS. The UnRS contains 18,269 genes (66.1% of the GenRS) and includes all the phosphoproteins present in the ExpRS plus those present in PredRS (Fig. 1). The IDs of all of these genes are available in Table S1.

### UnRS as a phosphoproteome reference set

**Fig. 2:**
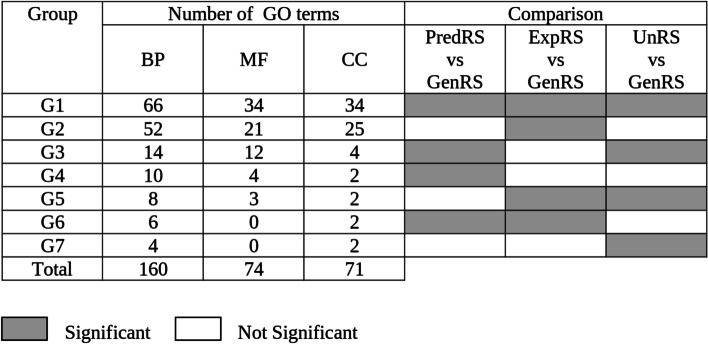
Heatmap representing significantly overrepresented GO terms in the phosphoproteome reference sets compared to the GenRS. Heatmaps represent the number of GO terms that change significantly in one, two or the three comparisons among the ExpRS, PredRS and UnRS phosphoproteome reference sets against the GenRS. Results are categorized in 7 groups (G1-G7). Gray: significant Parent-Child Fisher_FDR < 0.01; white: not significant. BP: Biological Process, MF: Molecular Function, CC: Cellular Component

In order to 1) evaluate if the frequency distribution of the GO terms in a phosphoproteome is equal to the frequency distribution in the genome and 2) evaluate the best phosphoproteome reference set, we performed GO annotation enrichment analysis using TopGO comparing the three reference phosphoproteomic datasets (ExpRS, PredRS and UnRS) against GenRS for the three ontologies. The results were plotted as binary heatmaps in which gray cells represent significant (Parent-Child Fisher_FDR < 0.01), and white cells non-significant GO terms for each comparison (PredRS vs. GenRS; ExpRS vs. GenRS and UnRS vs. GenRS) (Fig. 2). We focused our analyses in overrepresented GO terms. The terms assigned to the same heatmap’s pattern were categorized in groups (G1-G7) (Fig. 2). Groups were sorted according to the number of GO terms in each group for BP. G1 corresponded to the GO terms that were statistically different in all three comparisons, while the other groups could be arranged by complementary pairs (G2 and G3; G4 and G5; G6 and G7) (Fig. 2). In total, we found 160, 74 and 71 GO terms for BP, MF and CC, respectively, which behave significantly differently for one, two or the three comparisons (Fig. 2).

The GO enrichment analysis showed that most of the terms changed significantly in the three reference sets against GenRS (G1: 66, 34 and 34 terms for BP, MF and CC respectively) (Fig. 2), suggesting that these terms are enriched in the phosphoproteome datasets by default, independently of the condition or background. As it was expected, we found enrichment for the terms: “phosphorylation” (GO: 0016310) and “protein phosphorylation” (GO: 0006468) for BP and “protein kinase activity” (GO: 0004672), “transferase activity, transferring phosphates” (GO: 0016772), “phosphotransferase activity, alcohol group as acceptor” (GO: 0016773), “kinase activity” (GO: 0016301), and “purine ribonucleoside triphosphate binding” (GO:0035639) for MF (Table S2). Interestingly, we also found several terms enriched, but not direct descendant (child term) from a parent term involved in phosphorylation activities such as “reproduction” (GO:0000003), “reproductive process” (GO:0022414), “nitrogen compound metabolic process” (GO:0006807) and “organelle organization” (GO:0006996) for BP and “carbohydrate derivative binding” (GO: 0097367) and “ion channel activity” (GO:0005216) for MF (Table S2).

The second and third groups with the highest number of terms significantly enriched corresponded to G2 and G3 (Fig. 2 and Table S2). These groups form a pair of complementary groups in which the terms in PredRS vs. GenRS and UnRS vs. GenRS behave similarly to each other but differently from ExpRS vs. GenRS (Fig. 2). These differences could be accounted for by the 5132 phosphoproteins present in PredRS and UnRS but absent in the ExpRS. On the other hand, G4 and G5 form a pair of complementary groups in which the terms in ExpRS vs. GenRS and UnRS vs. GenRS behave similarly to each other but differently from PredRS vs. GenRS (Fig. 2 and Table S2). The terms in these groups could be accounted for by the 1113 phosphoproteins present in ExpRS and UnRS but absent in PredRS.

Interestingly, also only a few terms were found in G6 and G7 (10 terms out of 160 for BP, none of 74 for MF and 4 terms out of 71 for CC), that represent the groups in which the terms behave similar in ExpRS and PredRS datasets but different from UnRS (Fig. 2 and Table S2). In other words, when the terms in ExpRS and PredRS show the same behavior (both significantly different or not from the GenRS), only a few categories showed the inverse behavior in the UnRS. Thus, neither ExpRS nor PredRS contain the universe of phosphoproteins, and apart from having clear strengths previously mentioned, these datasets also display some disadvantages. On the one hand, in ExpRS the assembly of interpreted PTM using mass spectrometry in each study has many warnings that can lead to the incorrect data interpretation and wrong conclusions [16] and becomes relevant as the number of analyses in an assembled dataset increases [17]. On the other hand, as algorithms are trained for prediction with the experimental data available, PredRS may not be totally independent from ExpRS. Thus, neither is better than the other to be used as a reference set. In this regard, among the three, UnRS is the best reference set as it covers the largest amount of phosphoproteins and represents a low cost of GO terms that behave differently when ExpRS and PredRS are consistent in this regard. All these results suggest that UnRS is a plausible phosphoproteome reference set to be used to perform GO analysis.

In summary, GO term analysis of ExpRS, PredRS or UnRS revealed that several GO terms were enriched by default for BP, MF and CC with respect to the GenRS. So, when performing GO term enrichment analysis of a phosphoproteomic sample set compared only to GenRS, we obtain information about all the terms that changed in the sample but it is not possible to discern which of those terms changed specifically due to the treatment or background from those enriched by default. In this regard, we propose that additional comparison of the sample set with the UnRS (the best phosphoproteomic reference set) will uncover those terms that are specific to the experiment.

### Case study to uncover specific GO terms: phosphoproteome of etiolated Arabidopsis seedlings

Seedlings germinating in complete darkness follow a skotomorphogenic developmental program called etiolation. Etiolation is a critical phase during the life cycle characterized by an elongated hypocotyl and the formation of an apical hook that protects unopened cotyledons (without plastid differentiation) and shoot apical meristem when seedlings push through the soil on their way to the surface. To identify specific processes regulated by phosphorylation during etiolation, we performed a large-scale phosphoproteomic analysis of Arabidopsis etiolated seedlings (Et). We identified 933 unique phosphoproteins in phosphopeptide enriched samples from 5-days-old dark-grown seedlings that contain 2884 phosphosites (Table S3). These results are similar in number of phosphoproteins and phosphopeptides to other phosphoproteomic experiments [18, 19].

In order to evaluate the GO terms specifically enriched during skotomorphogenic development we performed GO enrichment analysis of the 933 phosphoproteins quantified in the Et Arabidopsis phosphoproteome against GenRS and UnRS. We found that from the 933 phosphoproteins identified experimentally in Et sample, 22 were new phosphoproteins never reported as such before (not included in ExpRS, Table S1). From these, 11 were present in UnRS (this means they could be predicted), but the other 11 were not present in the UnRS (this means that these phosphoproteins could not be predicted with the strict parameters used in PhosPhAt and MusiteDeep and have not been found in any previous phosphoproteome experiment available before) (Table S1). To perform GO term analysis, these new phosphoproteins are not a problem when the sample is compared against the GenRS because the last one contains all the genes in the genome. However, this may not be the case when the comparison is against the UnRS because this is a database that should always include all phosphorylated proteins. In other words, UnRS requires to be updated with new phosphoproteins found experimentally. So, we added our 11 new phosphoproteins to UnRS to define the Union+ 11 reference set (Un11RS) (Table S1). This set now contains 18,280 phosphoproteins. As it was expected for the addition of a reduced number of proteins, the UnRS and Un11RS differ only in 1 GO term for CC (Plastid membrane, GO: 0042170) when each one is compared to the GenRS.

**Fig. 3:**
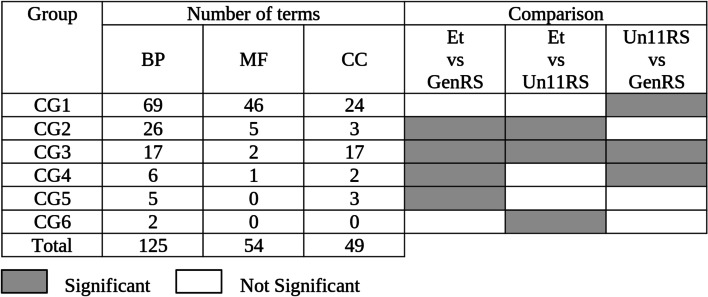
Heatmap representing significantly overrepresented GO terms in the Et phosphoproteome sample. Heatmap represents the number of GO terms that change significantly in one, two or the three comparisons: Et vs GenRS; Et vs Un11RS and Un11RS vs GenRS. Results are categorized in 6 groups (CG1-CG6; CG: for Contrast Group). Gray: significant Parent-Child Fisher_FDR < 0.01; white: not significant. BP: Biological Process, MF: Molecular Function, CC: Cellular Component

We then performed GO term enrichment analysis in Et samples by comparing Et vs. GenRS, Et vs. Un11RS and Un11RS vs. GenRS (Fig. 3). The latter is virtually a repetition of UnRS vs GenRS (Fig. 2). The results were plotted as binary heatmaps in which gray cells represent significant (Parent-Child Fisher_FDR < 0.01), and white cells non-significant GO terms for each comparison (Fig. 3). The terms assigned to the same heatmap’s pattern were categorized in six groups (CG1-CG6, CG for Contrast Group) (Fig. 3). Contrast groups were sorted according to the number of terms in each group for BP. We found 125, 54 and 49 terms for BP, MF and CC respectively that behave significantly differently for one, two or the three comparisons (Fig. 3, Table S4).

**Fig. 4:**
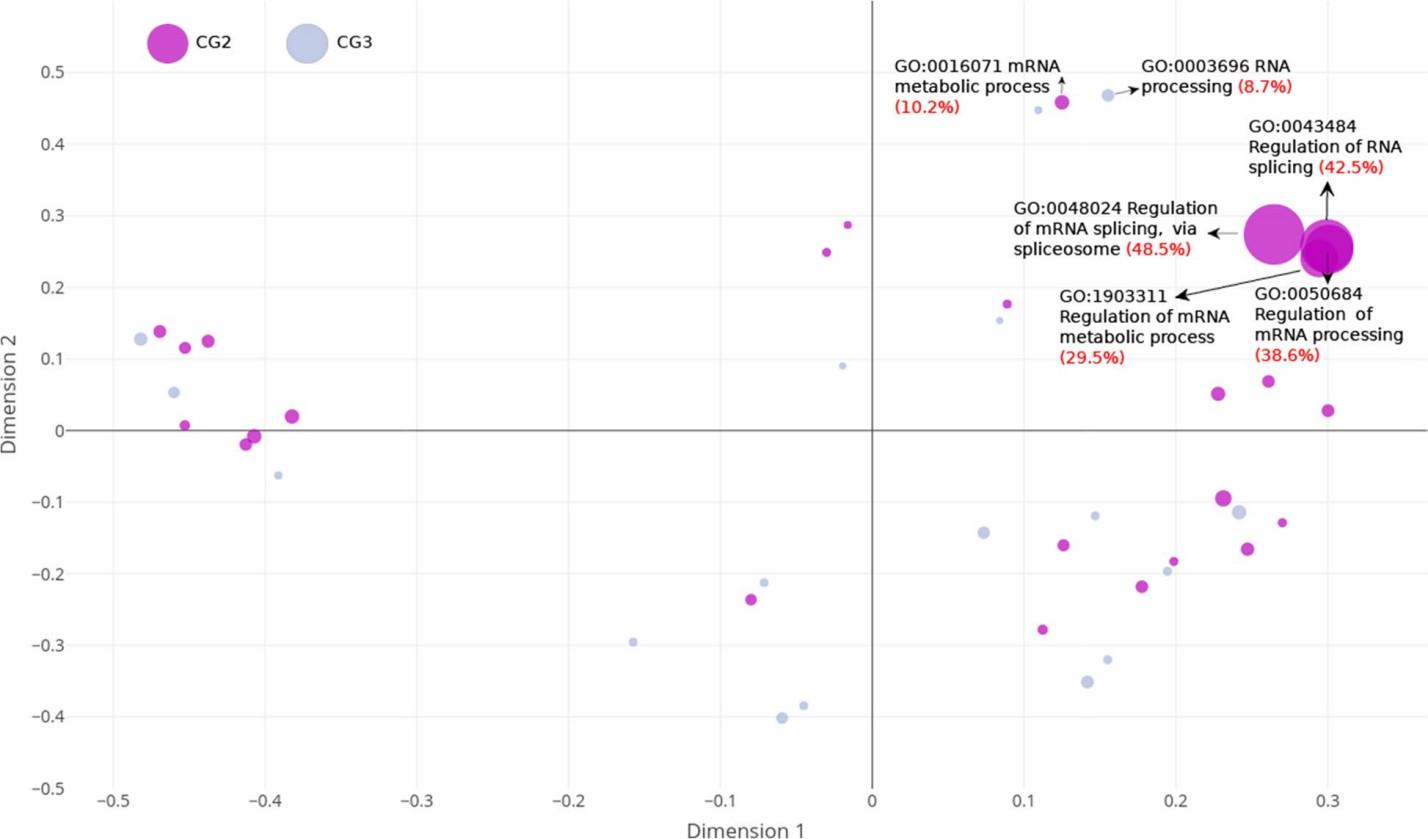
Semantic similarity for CG2 and CG3 for BP. The terms were represented graphically in two dimensions such that the distance between points on the plot approximates their multivariate dissimilarity as closely as possible. Each circle represents a GO term. The GenRatio is directly proportional to the diameter of the symbol and is indicated between brackets in red

According to our previous analysis we reasoned that a specific GO term is enriched in the Et sample when the GO term is significantly enriched in both comparisons: Et vs. GenRS and Et vs. Un11RS. Two groups fulfilled this statement: CG2 and CG3 (Fig. 3). The overrepresented terms in CG2 were significantly enriched in both comparisons (Et vs. GenRS and Et vs. Un11RS) but were not enriched in Un11RS (Un11RS vs GenRS, not significant). The terms in CG3 changed their frequency in all the comparisons, that means that their frequency in Et vs Un11RS increased beyond that in the already existing enrichment in Un11RS vs GenRS (Fig. 3). In other words, even though frequency distributions of the GO terms in CG3 are overrepresented in the phosphoproteome dataset with respect to the genome, these frequencies are even higher in the Et sample. Because different enriched GO terms can be related to each other and altogether highlight specific processes of relevance we further analyzed those specific GO terms more related to each other. For this, we investigated the topology similarity among GO terms in CG2 and CG3 by measuring the semantic similarity for each one of the ontologies. The semantic similarity is a measure of the interrelation between points [20]. The measures of semantic similarity allow us to obtain a numeric value according to the closeness of the significance of a term in a certain ontology. Thus, the more semantic similarity, the more closeness of the terms and the more functional similarity. On the other hand, we also calculated the GenRatio as the percentage of the genes associated with each GO term that are present in Et sample for CG2 and CG3 and for the three ontologies (GenRatio) (Table S5). Figure 4 shows a graphic representation of the semantic similarity among GO terms for BP where the diameter of the symbol is directly proportional to the GenRatio. Most of the GO terms for BP showed a GenRatio between 4 and 12% (Table S5). Surprisingly, this percentage rose up to 48.5% for the GO term “regulation of mRNA splicing, via spliceosome” (GO:0048024) and to 42.5% for “regulation of RNA splicing” (GO:0043484) (Fig. 4 and Table S5). Also, the terms “regulation of mRNA processing” (GO:0050684) and “regulation of mRNA metabolic process” (GO:1903311) showed 38.6 and 29.5% of GenRatio respectively (Fig. 4 and Table S5). The terms “mRNA metabolic process” (GO:0016071, 10.2%) and “RNA process” (GO:0006396, 8.7%) were also overrepresented GO terms specific to the Et sample although with lower GenRatios (Fig. 4 and Table S5). The relationships among these six GO terms are shown as a DAG (directed acyclic graph) (Fig. S1). Eighty-one unique phosphoproteins from Et sample were found to be present in the six GO terms related to RNA process and splicing in BP (Table S6). The specific terms “clathrin binding” (GO:0030276 29.7%), “protein serine/threonin/tyrosin kinase activity” (GO:0004712 18.6%) and “mRNA binding” (GO:0003729 16%) were the terms with the highest GenRatio for MF (Fig. S2 and Table S5). The terms “cytoplasmic microtubules” (32.3%), “nuclear membrane” (20%), “nuclear body” (18.6%) and spliceosomal complex (16%) present the highest GenRatios for CC (Fig. S2 and Table S5).

The group that contained more overrepresented terms was CG1 (69, 46 and 24 for BP, MF and CC respectively, Fig. 3). In this group, we found those terms only enriched in the comparison Un11RS vs. GenRS (the phosphoproteomic reference set), but not in the comparisons of the Et vs GenRS or Un11RS (Fig. 3 and Table S4). This group included those GO terms that were enriched in the phosphoproteome *per se*, but they were not enriched in the sample (phosphorylation pathways that do not participate during etiolation). CG4 represents the terms that were enriched in Et with respect to the GenRS, however these terms were not specific to the Et sample (Et vs Un11RS, not significative) rather to the phosphoproteome* per se* (Un11RS vs GenRS, significative) (Table S4). These could be genes associated with general, but not specific, phosphorylation signal processing also recruited to mediate dark signaling. CG5 and CG6 represent those GO terms that were significantly enriched only in the comparison of Et samples against GenRS or Un11RS respectively (Fig. 3 and Table S4). Both groups are formed by a small number of GO terms and all of them showed high parent-child _FDR values (Table S4).

In summary, a large-scale phosphoproteomic assay from etiolated seedlings identified 22 new phosphoproteins. Moreover, the implementation of a strategy to uncover specific phosphoproteomic pathways revealed that phosphorylation of proteins related to mRNA processing, and more specifically proteins involved in mRNA splicing is an important process that takes place during etiolation in Arabidopsis. Finally, “clathrin binding” for MF and “cytoplasmic microtubule” for CC were GO terms specifically enriched in etiolated seedlings.

## Discussion

One goal of almost all the phosphoproteomic studies is to uncover biological or signaling processes that are specific to an experimental condition or genetic background by analyzing the complete set of phosphoproteins identified. In this regard, GO term analysis is the most used bioinformatic tool. A key prerequisite for performing GO enrichment analysis is the availability of an appropriate reference set to compare against when looking for overrepresented terms. Generally, the whole genome is used as a reference set. However, when we compare phosphoproteome data against the genome it is assumed that the frequency distribution of the GO terms of a whole phosphoproteome is not different from the genome, a hypothesis not tested before. As a comprehensive phosphoproteome database is not available, we constructed three phosphoproteome reference sets: ExpRS, PredRS and UnRS from Arabidopsis. Our results show that GO term frequency in these phosphoproteome reference sets are statistically different from the GenRS, thus those GO terms are enriched in the phosphoproteome of Arabidopsis by default (Fig. 2, Table S2)*.*

In this work, we provide evidence that UnRS is a plausible and the best set to be used as a phosphoproteome reference set that would help in uncovering those GO terms specific to an Arabidopsis phosphoproteome sample. When PredRS and ExpRS behave similarly in their GO term frequency only a few terms present the opposite pattern in UnRS (Fig. 2). As we show here, it is important to update UnRS as new phosphoproteomic experiments are performed.

We show and characterize the phosphoproteome of etiolated Arabidopsis seedlings, identifying 933 proteins that are phosphorylated in 5-days-old dark-grown seedlings (Table S3). Twenty-two of those phosphoproteins have not been reported before, 11 of which could not be predicted either (Table S1). Thus, we updated UnRS with these 11 phosphoproteins to obtain Un11RS and use it as a reference set.

Our results show that in a standard comparison of the Et sample to the Genome only (Et vs GenRS), we can find 54, 8 and 25 significantly overrepresented GO terms for BP, MF and CC respectively (sum of the terms in groups CG2 to CG5, Fig. 3). However, of these, 11, 1 and 5 terms for BP, MF and CC respectively are predicted not to be specific to the phosphoproteome of etiolated tissues (sum of the terms groups CG4 and CG5, Fig. 3). Using additional comparison of Et sample with Un11RS enables us to uncover specific GO terms related to RNA processing, and more specifically mRNA splicing for the three ontologies (Fig. 3, Fig.4, Table S5 and Fig. S2). These terms were not only specifically overrepresented in Et samples, but also they showed the highest GenRatios rising up to 48.5% (Fig. 4, Fig. S2 and Table S5). The GO terms “cytoplasmic microtubule” for CC and “clathrin binding” for MF were also over represented with high GenRatios (Fig. S2) and could be related to active cellular expansion taking place during etiolation.

In RNA splicing, the accurate process of the precursor-mRNAs affects mRNA stabilization regulating different developmental programs. In particular, genes containing introns undergo alternative splicing leading to multiple mRNAs. The Arabidopsis SR (serine-arginine rich) protein family is a highly conserved family of RNA binding proteins involved in the regulation of precursor-mRNA splicing. In this study, we found 7 SR genes: SR34, RSZ22a, SC35, SCL30, RS2Z33, RS31, RS40 and RS41 (Table S6). Notably, SCL30, RS2Z33, RS31, RS40 and RS41 were among the ten plant specific SR genes [21], suggesting that phosphorylation and the molecular mechanism of SR proteins in etiolated seedlings might be unique.

It is well known that many proteins involved in splicing are regulated by phosphorylation [3, 22]. PRP4KA (PRE-MRNA PROCESSING 4 KINASE A) is a spliceosome-associated kinase in Arabidopsis that affects alternative splicing patterns by phosphorylating different splicing target regulators [23]. Interestingly, we found PRP4KA as a phosphorylated protein in the Et sample (AT3G25840, Table S6). More surprisingly, we found 7 targets of PRP4KA phosphorylation: 3 splicing factors (ATSF1, RS40 and RS41), 3 RNA binding proteins (BTR1L, CCR2 and ELF9) and 1 mRNA decapping protein (VCS) (Table S6 and [23]. We found PRP40A (PRE-MRNA-PROCESSING PROTEIN 40A) and PRP40B (PRE-MRNA-PROCESSING PROTEIN 40B) in Et samples, two phosphoproteins that bind the phosphorylated C-terminal domain of the largest subunit of RNA polymerase II. SR proteins can be recruited to the RNA polymerase II complex interacting by its carboxy terminal domain [24], and its phosphorylation plays a major role in transcription that finally regulates development and growth.

Our results show that phosphorylated RNA splicing proteins are highly overrepresented in dark tissues and pre-mRNA processing by alternative splicing might be an important co-transcriptional process relevant during etiolation development. Transition to light also changes the alternative splicing of several genes in etiolated and light-grown Arabidopsis [25, 26]. From the 81 phosphoproteins in Et samples related to RNA processing and splicing, we found 25 which alternative splicing changes upon light treatments (Table S6). In this context, phosphorylation patterns of RNA splicing proteins in the dark might not be trivial then for the de-etiolation process. How the phosphorylation status of splicing related proteins is important for etiolation or for light to trigger a rapid response to de-etiolation is an interesting matter for future studies.

The term “cytoplasmic microtubules” was highly overrepresented in the etiolated phosphoproteome (Fig. 2). This term includes phosphoproteins that bind and modulate cytoplasmic microtubule orientation called MAPs (Table S6). Phosphorylation is a well-known regulation mechanism in MAPs [27]. We found MAP65-1 and MAP65-2 among the 10 phosphoproteins in the term “cytoplasmic microtubule” (Table S6), that have been reported to promote hypocotyl elongation in dark [28]. WDL3 was also a phosphoprotein found in the term cytoplasmic microtubules. In the dark, the MAP WDL3 is recognized by its phosphorylation pattern and degraded by the 26S proteasome, while in light WDL3 is stabilized favoring the longitudinal orientation of microtubules arresting hypocotyl growth [29]. These results suggest that phosphorylation of MAPs as a whole is important during etiolation. The relationship between MAPs phosphorylation and hypocotyl elongation in dark requires more investigations.

It is noteworthy that the 69, 46 and 24 terms for BP, MF and CC respectively from CG1 (Fig. 3) highlight GO terms involving phospho-signaling that do not have a relevant role in dark development. We found terms related to defense response for BP and plastid and thylakoid for CC in this group (Table S6). This is consistent with the hypothesis that plants invest its resources in functions that comprise its fitness rather than in other functions as defense that could be inducible when they are needed [30]. In the dark, plastids and thylakoids are not even developed.

## Conclusions

In this work, we show a novel strategy that includes the comparisons of a phosphoproteome sample not only against the genome but also against a phosphoproteome database of reference. We propose UnRS, a database containing all the phosphoproteins found experimentally plus those that could be predicted with strict parameters, as the phosphoproteome reference set in Arabidopsis. The only requirement to perform this strategy is to update the database with the new phosphoproteins that have been identified. This novel strategy has the power to single out, from an array of phosphorylation-activated processes, those that are specific for a particular condition. Additionally, this strategy could be applicable to other species with public available phosphoproteome data. We also show that RNA splicing, microtubule and clathrin binding proteins are promising areas of research in dark development in Arabidopsis as major targets of phosphorylation.

## Methods

### Procedures for building Phosphoproteomes of reference

#### GenRS

To obtain the list of protein-coding genes, the whole *Arabidopsis thaliana* proteome was downloaded from arabidospis.org website (Data Sources). This list is composed of 48,359 proteins since every splicing variant is included, and they are associated with in-use IDs (Table S7 (see S7.1)). So, it became the reference list to map every other dataset to get rid of obsolete IDs. After trimming the splicing variants and filtering for unique registers, a list of 27,655 root-IDs were retrieved (Table S7 (see S7.2)), that correspond to all *Arabidopsis thaliana* protein-coding genes [15]. This dataset is GenRS (Table S1, Table S7 (see S7.3)).

#### ExpRS

To build ExpRS, 55 of the most significant *Arabidopsis thaliana* phosphoproteomic datasets published in literature were collected. These datasets were mostly retrieved from the updated databases PhosPhAt4.0 [31–33] and P3DB3.5 [14] and supplemented with [8] data; and the recent released *Arabidopsis thaliana* expression atlas which contains one of the most comprehensive single *Arabidopsis thaliana* phosphoproteomes published so far [9]. Links to databases can be found at Data Sources. Among all these studies included; 17 used cell cultures, and the rest used soil-, plate-, or hydroponically grown tissue, including young seedlings and leaves/rosettes (32), roots (6), pollen (2), or seeds (5). Various types of stress treatments were employed, in particular nutrient stress (nitrogen, sugars, and phosphate), but also hormone treatments (e.g., ethylene and abscisic acid) and biotic stress (as exposed previously in [8]). However, more than 65% of the ExpRS dataset belongs to control conditions. To assemble ExpRS, we retrieve the lists of genes encoding for phosphoproteins identified by mass spectrometry in each study. All in all, ExpRS contains 13,137 genes that encode for proteins with experimental evidence of phosphorylation (Table S1, Table S7 (see S7.4)).

#### PredRS

The predicted phosphoproteome named PredRS, was built combining two of the most accurate and updated predictors used in plants: MusiteDeep [34–36] and PhosPhAt [31–33]. We evaluated performance of MusiteDeep, PhosPhAt and the ensemble of both classifiers (PredRS), in order to achieve a high confident prediction with the highest number of phosphoprotein-coding-genes experimentally validated (Table S7 (see S7.5)).

#### MusiteDeep

MusiteDeep [34–36] provides a deep-learning framework for protein post-translational modification site prediction [34]. The method overview and deep learning architecture are well detailed in [34].

MusiteDeep phosphorylation predictor uses protein sequences as input and calculates a score at the amino acid level. All the residues annotated by UniProtKB/Swiss-Prot as phosphorylated were treated as positive sites, while the residues with the same amino acids excluding annotations were regarded as the negative sites [34].

With the aim to formulate the prediction as a binary classification between “gene encoding for a phosphorylated protein” or positive result, and “gene encoding for a non-phosphorylated protein” or negative result; a prediction score was required to be assigned per protein rather than per site. In this regard, we considered a positive prediction if MusiteDeep predicted any site as phosphorylated. This constituted a new classifier based on MusiteDeep.

All the Arabidopsis proteins’ sequences were submitted to MusiteDeep Phosphorylation (S,T) and Phosphorylation (Y) prediction models (Data Sources). The cutoff was set to zero in order to obtain the complete unfiltered set of putative phosphoserine S, phosphothreonine T and phosphotyrosine Y) (Table S7 (see S7.6)). For every S, T and Y in each protein, a prediction score was obtained (Table S7 (see S7.7)), otherwise we setted − 1 as a flag for missing prediction scores. We retrieved the maximum prediction score among phosphoserine score, phosphothreonine score and phosphotyrosine score for each protein (Table S7 (see S7.8)). We then calculated a single score for each protein (protein score) as the maximum between phosphoserine, phosphothreonine and phosphotyrosine scores (Table S7 (see S7.9)). As exposed previously, several proteins may be encoded by the same gene due to splicing variants. So we calculated a prediction score (Gene Score) for each gene as the maximum of protein scores within the proteins encoded by the same gene (Table S7 (see S7.10)). These gene scores belong to the new classifier based on MusiteDeep.

A true positive result (tp) is a gene predicted as coding for a phosphorylated protein and included in ExpRS, thus experimentally validated. In this regard, each gene has been labeled according to ExpRS so as to judge it as a true or false positive prediction. According to this, a false positive result (fp) is a gene predicted as coding for phosphorylated protein and not included in ExpRS. A true negative result (tn) is a gene predicted as coding for non-phosphorylated protein and not included in ExpRS. A false negative result (fn) is a gene predicted as coding for non-phosphorylated protein and included in ExpRS. These parameters characterize a classifier’s performance.

To determine the optimal classifier’s threshold for gene scores, we have calculated the value on the ROC curve (Receiver Operating Characteristic) which maximizes true positive rate (tpr = tp / (tp + fn)) and minimizes false positive rate (fpr = fp / (fp + tn)). AUC (Area under ROC Curve) score for this classifier was 0.73 and the value obtained for the threshold was 0.85. Therefore, the optimal threshold for this classifier was calculated as the mean of the results from computing the optimal threshold value for 5000 random samples of 30% of the total set. With threshold set in 0.85, classifier’s performance results in fpr = 0.35, tpr = 0.69 and 4105 genes predicted as fn.

#### PhosPhAt

PhosPhAt offers a phosphorylation site prediction tool specifically trained on experimentally identified *Arabidopsis thaliana* phosphorylation motifs [14]. It is based on a Support-Vector-Machines algorithm whose feature-vector consists of the sequence of amino acids and their chemical-physical properties [14]. The high-confident predicted sites are available to download from PhosPhAt website (Data Sources).

We retrieved the genes that encode for proteins with predicted sites by PhosPhAt, and considered as true positive results every gene included in ExpRS. According to this, PhosPhAt’s performance resulted in fpr = 0.001, tpr = 0.769 and 3034 genes predicted as fn.

#### PredRS performance

PredRS resulted from the combination of PhosPhAt and MusiteDeep predictions. PredRS considered a gene as encoding for a phosphorylated protein, if either PhosPhAt or MusiteDeep predicted it as positive.

Metrics obtained for PredRS performance were the following: Sensitivity or recall = 0.91, specificity = 0.66, precision = 0.71, fpr = 0.34, tpr = 0.91 and 1113 genes predicted as fn.

According to our evaluation of the predictors’ performance on Arabidopsis data, PhosPhAt outperformed MusiteDeep, because of lower fpr and higher tpr. However, PredRS presented the highest tpr and lowest fn, leading to high sensitivity and precision values. This is the reason why it constituted the predicted phosphoproteome of reference in this study. PredRS contains 17,156 highly confident predicted phosphoprotein-coding genes (Table S1 and Table S7 (see S7.11)), 70% of which are experimentally validated (intersect with ExpRS).

#### UnRS

To assemble the current *Arabidopsis thaliana* phosphoproteome so far, we performed the union of ExpRS and PredRS, leading to a list of 18,269 phosphoprotein-coding genes (Table S7 (see S7.12)). For contrast tests, 11 novel phosphoproteins from in-house unpublished data were added to UnRS becoming U11RS (Table S1).

### Phosphoproteomic from etiolated Arabidopsis seedlings

#### Plant material

WT seeds from ecotype *Landsberg erecta* background were obtained from the *Arabidopsis Biological Resource center* (ABRC, Ohio, USA). No specific permission is need to use WT ABRC seeds. Seeds were sterilized for 2 h in a Cl_2_ (g) atmosphere generated by the addition of 1.5 ml HCl (37% v/v) to 50 ml of bleach (sodium hypochlorite). Sterilized seeds were sown on Murashige-Skoog 0.5X agar 0.8% (w/v) medium in petri dishes and incubated at 4 °C in D during 3 d for stratification. Chilled seeds were exposed to a 2 h red light pulse (50 μmol m^− 2^ s^− 1^) at 22 °C to synchronize germination, and then kept in darkness for 5 d before harvest.

### Protein extraction, phosphopeptide enrichment and LC-MS/MS

Fifteen g of seedlings from 5-d-dark-grown WT seedlings were harvested and grinded with mortar and pestle in liquid N_2_. Seedling powder was resuspended in 750 μl of cold extraction buffer (0.7 M sucrose, 0.1 M KCl, 0.5 M Tris-Cl, pH 7.5, 50 mM EDTA and 2% v/v β-Merchaptoethanol). Seven hundred fifty microliters of phenol equilibrated at pH 8 with Tris-HCl was added and incubated at 4 °C for 30 min. Phases were separated by centrifugation during 30 min at 9000 *g* (4 °C). The phenolic phase was recovered and re-extracted with an equal volume of cold extraction buffer. Proteins were precipitated by addition of five volume of 0.1 M ammonium acetate in methanol (chilled at − 20 °C) to the phenolic phase, incubated overnight at − 20 °C, and centrifuged during 1 h at 12,000 *g* (4 °C). The pellets were washed twice with two volumes of cold methanol. The washing procedure was repeated twice with two volumes of cold acetone. The pellets were dried and resuspended in 100 μl of resuspension buffer (50 mM potassium phosphate buffer, pH 7.5, 8 M urea, protease inhibitors 1X (RocheTM) and phophatase inhibitors 1X (RocheTM). Total proteins were quantified by Bradford method [37] and lyophilized. Lyophilized proteins were sent for phosphopeptide enrichment and mass spectrometry label free quantification to the Proteomic Platform of Chu de Quebec Research Centre (Laval, QC). Samples were resuspended in 450 μl of 50 mM Ammonium bicarbonate. A trypsin digestion of 450 μg of each sample was performed followed by phosphopeptide enrichment using Pierce™ TiO_2_ Phosphopeptide Enrichment and Clean-up Kit. 1/5th of the elution for each sample was injected in a Thermo Orbitrap Fusion Mass Spectrometer. It was performed a 120 min run with a 90 min gradient in a data dependent acquisition (DDA) with high energy collision induced dissociation (HCD) MS/MS-IT detection mode. We performed 3 independent replicates.

#### Bioinformatic analysis

The bioinformatic analysis was performed using MaxQuant/Andromeda engine, setting as fixed modifications: Carbamidomethylation (C), and variable modifications: Oxidation (M) + Phosphorylation (STY). The database employed was UniProt CP_Arabidopsis Thaliana. The quantification was done using signal intensity values of peptides. A peptide was considered quantifiable if it had at least 2 signal intensity values among biological replicates. Missing signal intensity values were imputed with a noise value corresponding to the 1-percentile of all intensity values by sample. The mass spectrometry proteomics data have been deposited to the ProteomeXchange Consortium via the PRIDE [38] partner repository with the dataset identifier PXD008274.

This in-house data corresponds to the etiolated Arabidopsis seedlings’ phosphoproteome, called Et in this study. It contains 933 genes that encode for phosphoproteins quantified in this study (Table S1), out of a total of 1152 identified filtering with FDR 1%.

### GO enrichment analysis in TopGO

Semi-automated Gene Ontology (GO) terms enrichment analysis [39] were performed through the TopGO package as described in [40]. We implemented and applied the ParentChild algorithm [41] for eliminating local similarities and dependencies between GO terms. For statistics, we computed Fisher’s exact test which is based on gene counts [42] (Table S7 (see S7.13)).

The GO is composed of thousands of functional classes (terms) structured according to a directed acyclic graph (DAG). When a gene is annotated with a class, all inferences emerging from the structure of the GO must also hold true. This is known as the “True Path Rule”. In other words, “an annotation for a class in the hierarchy is automatically transferred to its ancestors, while genes unannotated for a class cannot be annotated for its descendants” [43]. Provided a list of terms enriched in a gene dataset, if the child term has highly statistically significant enrichment, the parent term might appear significantly enriched purely as a consequence of including all the genes from the child term. This is the reason why GO enrichment analysis cannot be done independently for each term; if so, each gene would be counted multiple times.

To build the TopGO data object, three elements were required: the GO annotations for mapping the genes to the GO terms [44–46]; the dataset of reference to which every dataset was compared (gene universe or population); and the dataset to be tested for enrichment of GO terms (intersection with the gene universe must be complete). GO annotations for the three ontologies (Biological Process, Molecular Function and Cellular Component) were retrieved from GO.db version 3.12 (Data Sources). Mapping was performed as specified in Table S7 (see S7.14). GenRS was defined as the gene universe for comparing every phosphoproteomic dataset in this study (PredRS vs. GenRS, ExpRS vs. GenRS, UnRS vs. GenRS in Fig. 2; and Et vs. GenRS, Un11RS vs. GenRS in Fig.3). And Un11RS became the gene universe when comparing Et (Et vs. Un11RS in Fig. 3). Statistical analysis yielded *p*-values that were adjusted for multiple testing. In consequence, FDR correction procedure produced conservative p-values and declared fewer terms as significant. We considered significant those values smaller than 0.01 (Table S7 (see S7.15)).

A binary matrix was constructed with the significant (taking value 1) and non-significant (taking value 0) GO terms, for each comparison in Fig. 2 and Fig.3 (Table S7 (see S7.16)). These results were resumed as binary heatmaps, in which the GO terms sharing the same pattern were classified into groups. For each ontology, the count of GO terms in each group was specified.

### Semantic similarity analysis

In order to identify the phosphorylation pathways in Et, we determined the proportion of genes associated with every significant GO term in groups CG2 and CG3, that are present in Et sample; and their interrelation in the GO graph. First, we constructed the lists of genes associated with the significant GO terms in each group (Table S7 (see S7.17 and S7.18)). Then, we computed the GenRatio (Calculation of percentage of genes associated with each GO term that are present in Et sample) for each of these terms in groups CG2 and CG3. (Table S7 (see S7.19)). Finally, semantic similarity graphs were built in order to locate the significant terms in the GO structure using GenRatio values as nodes’ sizes (Fig. 4).

The semantic comparisons of Gene Ontology (GO) annotations provide quantitative ways to compute similarities between genes and gene groups [20]. Considering that the specificity of a GO term is usually determined by its location in the GO graph, it has been proposed a graph-based strategy to compute semantic similarity using the topology of the GO graph structure [47]. In Wang’s method, the semantics of GO terms are encoded into a numeric format and the different semantic contributions of the distinct relations are considered [20]. GO terms Semantic similarity was calculated using ViSEAGO package [48] (Table S7 (see S7.20)). The Wang semantic similarity plots display nodes scattered in a 2-dimension space according to their semantic similarity. Every node represents a significant GO term coloured according to the comparison/s, and their size represents their corresponding GenRatio. DAG graphs were built using GOview (Data Sources) so as to show the relationship of GO terms with higher GenRatios (Fig. S1).

## Supplementary Information

**Additional file 1 **: **Table S1.** IDs corresponding to all the datasets used in this study.

**Additional file 2 **: **Table S2.** Overrepresented GO terms IDs for BP, MF and CC from the different groups of Fig. 1.

**Additional file 3 **: **Table S3.** Phosphoproteome of 5 day old dark-grown Arabidopsis seedlings. The table contains information of the IDs, the phosphopeptide intensity for each repetition and the phosphosites.

**Additional file 4 **: **Table S4.** Overrepresented GO terms IDs for BP, MF and CC from the different contrats groups of Fig. 2.

**Additional file 5 **: **Table S5.** Percentage of the genes from Et sample present in the genes associated to each GO term (GenRatio) for CG2 and CG3 and for BP, MF and CC.

**Additional file 6 **: **Table S6.** IDs and descriptions of the 81 unique phosphoproteins from Et sample that were present in the six GO terms related to RNA process (BP) for CG2 and CG3 (Fig. 2).

**Additional file 7 **: **Table S7.** References to datasets and source code of each pipeline step generated in the present study.

**Additional file 8 **: **Figure S1.** DAG (directed acyclic graph) representing the relation among the six GO terms related to RNA process for BP.

**Additional file 9 **: **Figure S2**. Semantic similarity for the GO terms in CG2 and CG3 for MF and CC. Each circle represents a GO term. The GenRatio is directly proportional to the diameter of the symbol and is indicated between brackets in red.

**Additional file 10 **: **Data Sources**. Contains all the bioinformatics data sources used in this study.

## Data Availability

All data generated or analysed during this study are included in this published article and its supplementary information files, as well as in public open-access databases. All datasets used to build GenRS, ExpRS, PredRS and UnRS are open and available in the public repository https://github.com/paulati/arabidopsis_phospho/tree/master/data/raw. Links to referenced data sources and databases can be found at Data Sources file. The mass spectrometry proteomics data to build Et can be found at PRIDE [37] repository with the dataset identifier PXD008274. Source code for the construction of GenRS, ExpRS, PredRS and UnRS; TopGO enrichment analysis and semantic similarity analysis are available in the public repository https://github.com/paulati/arabidopsis_phospho. (References to datasets and source code of each pipeline step are available in Table S7).

## Abbreviations

BP: Biological Process
CG1-CG6: Contrast Groups 1–6
CC: Cellular Component
ExpRS: Experimental Reference Set
G1-G6: Group 1–6
GenRS: Genome Reference Set
GO: Gene Ontology
MF: Molecular Function
PredRS: Predicted Reference Set
PTM: Post Translational Modification
SR: Serine-arginine Rich
UnRS: Union Reference Set
Un11RS: Union plus 11 Reference Set
