# Supplementary material for: A novel strategy to uncover specific GO terms/phosphorylation pathways in phosphoproteomic data in Arabidopsis thaliana
Source: BMC Plant Biol. 2021 Dec 14;21:592. doi: 10.1186/s12870-021-03377-9 (PMC8670200; doi:10.1186/s12870-021-03377-9)
Supplement: Supplementary file 7 — Additional file 7 : Table S7. References to datasets and source code of each pipeline step generated in the present study. [file 12870_2021_3377_MOESM7_ESM.docx]

**Additional File 7. Table S7.**

**Datasets and Pipeline steps**

| S7.1 | Arabidopsis raw protein-coding genes list | <https://github.com/paulati/arabidopsis_phospho/blob/master/data/preproc/Araport11_genes_all.zip> |
| --- | --- | --- |
| S7.2 | Arabidopsis root-IDs list building | [https://github.com/paulati/arabidopsis_phospho/blob/master/preparation/1_all_proteins.ipyn](https://github.com/paulati/arabidopsis_phospho/blob/master/preparation/1_all_proteins.ipynb) |
| S7.3 | GenRS root-IDs list | <https://github.com/paulati/arabidopsis_phospho/blob/master/data/results_preproc/all_ids.zip> |
| S7.4 | ExpRS root-IDs list | <https://github.com/paulati/arabidopsis_phospho/blob/master/data/results_preproc/ExpRS.zip> |
| S7.5 | Predicted phosphoproteome root-IDs list building | <https://github.com/paulati/arabidopsis_phospho/blob/master/preparation/3_predictions.ipynb> |
| S7.6 | MusiteDeep phosphoproteome prediction scores | h[ttps://github.com/paulati/arabidopsis_phospho/blob/master/data/results_preproc/musitedeep/filenum_Prediction_results.txt.zip](https://github.com/paulati/arabidopsis_phospho/blob/master/data/results_preproc/musitedeep/filenum_Prediction_results.txt.zip) |
| S7.7 | MusiteDeep phosphoserine, phosphothreonine and phosphotyrosine scores by protein | (<https://github.com/paulati/arabidopsis_phospho/blob/master/data/results_preproc/musitedeep/Prediction_results_all.zip> |
| S7.8 | MusiteDeep max phosphoserine, phosphothreonine and phosphotyrosine scores by protein | <https://github.com/paulati/arabidopsis_phospho/blob/master/data/results_preproc/musitedeep/Prediction_results_all_scores.zip> |
| S7.9 | MusiteDeep based prediction score by protein | <https://github.com/paulati/arabidopsis_phospho/blob/master/data/results_preproc/musitedeep/Prediction_results_score_by_protein.zip> |
| S7.10 | MusiteDeep based prediction scores by root-ID | <https://github.com/paulati/arabidopsis_phospho/blob/master/data/results_preproc/musitedeep/Prediction_results_score_by_id_base.zip> |
| S7.11 | PredRS root-IDs list | <https://github.com/paulati/arabidopsis_phospho/blob/master/data/results_preproc/PredRS.zip> |
| S7.12 | [UnRS](https://github.com/paulati/arabidopsis_phospho/blob/master/data/results_preproc/UnRS.zip) root-IDs list | <https://github.com/paulati/arabidopsis_phospho/blob/master/data/results_preproc/UnRS.zip> |
| S7.13 | GO Terms analysis | <https://github.com/paulati/arabidopsis_phospho/blob/master/topgo/go_terms_analysis.R> |
| S7.14 | GO Annotations | <https://github.com/paulati/arabidopsis_phospho/blob/master/topgo/annotations.R> |
| S7.15 | topGO enrichment results | <https://github.com/paulati/arabidopsis_phospho/tree/master/data/results_topgo> |
| S7.16 | Binary matrix with significant and non-significant GO terms for each comparison | <https://github.com/paulati/arabidopsis_phospho/blob/master/data/results_topgo/fig2/fig2_go_terms_matrix.csv> |
|  |  | <https://github.com/paulati/arabidopsis_phospho/blob/master/data/results_topgo/fig3/fig3_go_terms_matrix.csv> |
| S7.17 | Building of lists of genes associated with each GO term in each group | <https://github.com/paulati/arabidopsis_phospho/blob/master/lists_by_group/go_terms_lists.R> |
| S7.18 | Lists of genes associated with each GO term in each group | <https://github.com/paulati/arabidopsis_phospho/tree/master/data/lists_by_groups> |
| S7.19 | Calculation of percentage of genes associated with each GO term that are present in Et sample | h[ttps://github.com/paulati/arabidopsis_phospho/blob/master/semantic_similarity/et_terms_perc_tables.R](https://github.com/paulati/arabidopsis_phospho/blob/master/semantic_similarity/et_terms_perc_tables.R) |
| S7.20 | Semantic similarity | <https://github.com/paulati/arabidopsis_phospho/blob/master/semantic_similarity/viseago_topGo_combinatory_fig4.R> |
