# Supplementary figures and images for: A novel strategy to uncover specific GO terms/phosphorylation pathways in phosphoproteomic data in Arabidopsis thaliana
Source: BMC Plant Biol. 2021 Dec 14;21:592. doi: 10.1186/s12870-021-03377-9 (PMC8670200; doi:10.1186/s12870-021-03377-9)

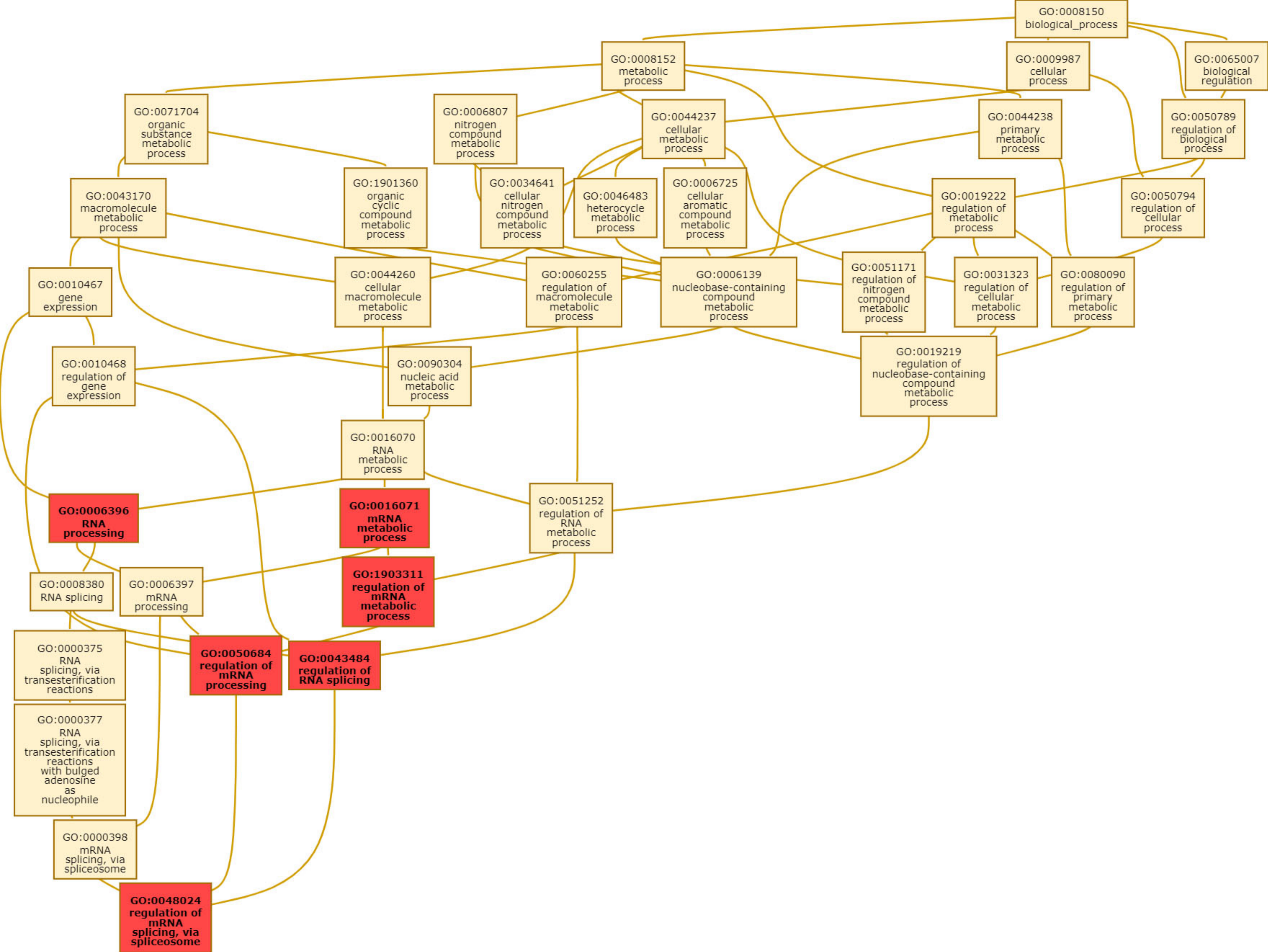

Supplement: Supplementary file 8 — Additional file 8 : Figure S1. DAG (directed acyclic graph) representing the relation among the six GO terms related to RNA process for BP. [file 12870_2021_3377_MOESM8_ESM.pdf]

MF

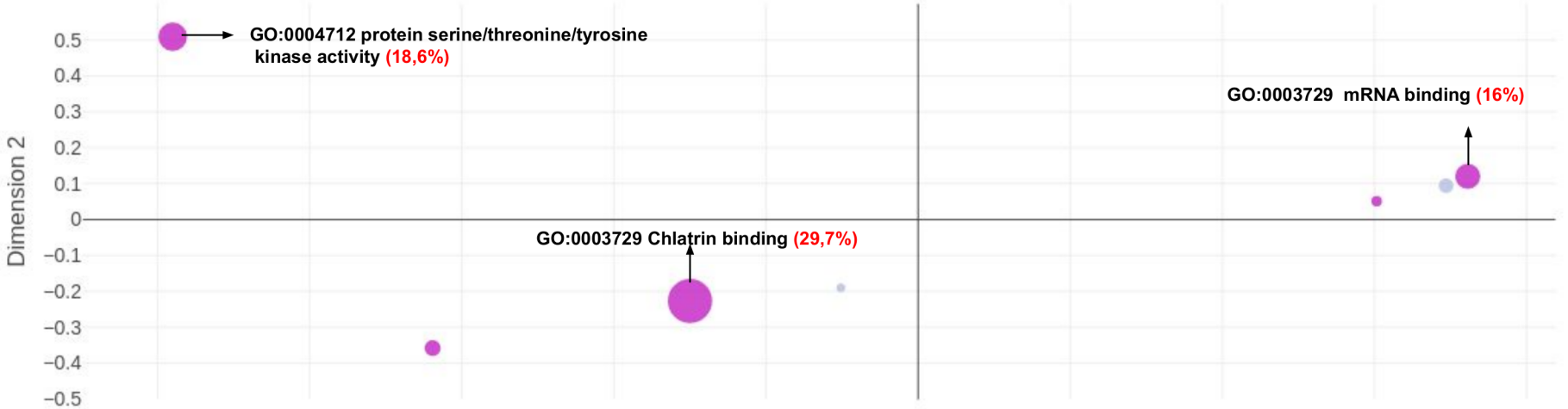

CC

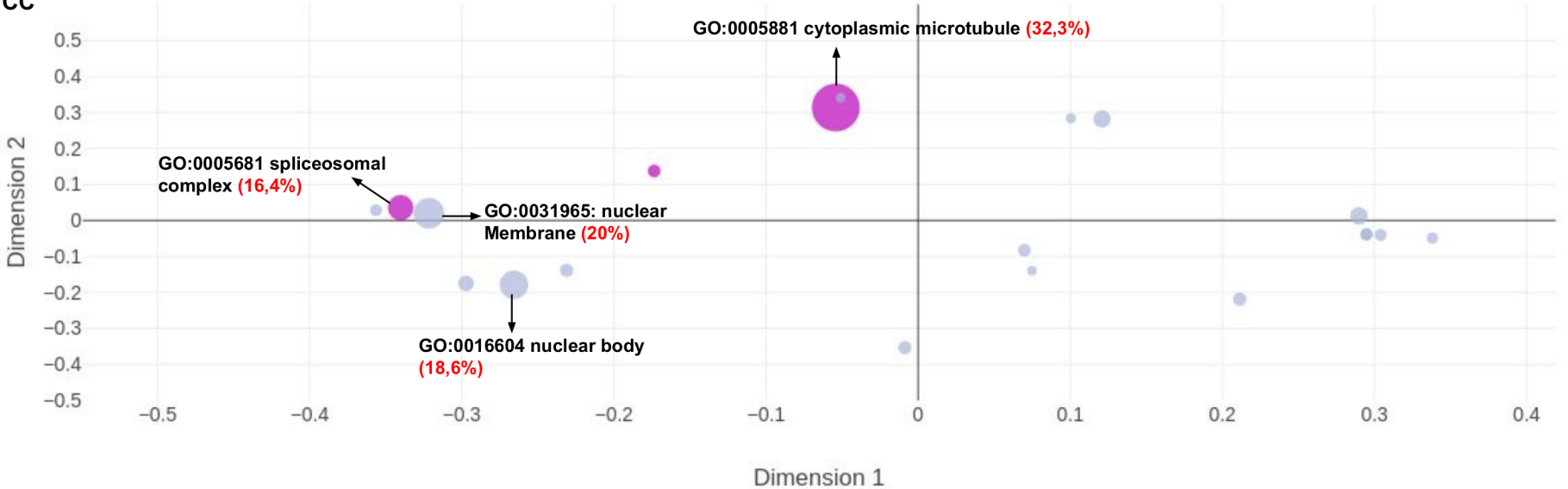

Supplement: Supplementary file 9 — Additional file 9 : Figure S2. Semantic similarity for the GO terms in CG2 and CG3 for MF and CC. Each circle represents a GO term. The GenRatio is directly proportional to the diameter of the symbol and is indicated between brackets in red. [file 12870_2021_3377_MOESM9_ESM.pdf]
