# Supplementary material for: A novel strategy to uncover specific GO terms/phosphorylation pathways in phosphoproteomic data in Arabidopsis thaliana
Source: BMC Plant Biol. 2021 Dec 14;21:592. doi: 10.1186/s12870-021-03377-9 (PMC8670200; doi:10.1186/s12870-021-03377-9)
Supplement: Supplementary file 10 — Additional file 10 : Data Sources. Contains all the bioinformatics data sources used in this study. [file 12870_2021_3377_MOESM10_ESM.docx]

| **Name** | **Type** | **Url** | **Last updated** | **Github** |
| --- | --- | --- | --- | --- |
| *Arabidopsis thaliana´s* proteome | data | <https://www.arabidopsis.org/download/index-auto.jsp?dir=%2Fdownload_files%2FProteins%2FAraport11_protein_lists> | March 8, 2021 | https://github.com/paulati/arabidopsis_phospho/blob/master/data/raw/Araport11_genes.201606.pep.fasta.gz |
| PhosPhAt4.0 | Experimental data | <http://phosphat.uni-hohenheim.de/Phosphat_20200624.csv> | June 24, 2020 | https://github.com/paulati/arabidopsis_phospho/blob/master/data/raw/Phosphat_20200624.zip |
| P3DB3.5 | data | <http://p3db.org> | 2020 | https://github.com/paulati/arabidopsis_phospho/blob/master/data/raw/p3db-3.5-phosphosite-report_Arabidopsis-thaliana.gz |
| van Wijk et.al., 2014 | data | <https://academic.oup.com/plcell/article/26/6/2367/6098503#234766096>  Supplemental Data /  Supplemental Data Set 2 |  | https://github.com/paulati/arabidopsis_phospho/blob/master/data/raw/SupplementalDataSet_2rfinal.zip |
| Mergner et.al., 2020 | data | https://static-content.springer.com/esm/art%3A10.1038%2Fs41586-020-2094-2/MediaObjects/41586_2020_2094_MOESM14_ESM.xlsx (ATHENA, athena.proteomics.wzw.tum.de; ProteomicsDB, www.proteomicsdb.org) |  | https://github.com/paulati/arabidopsis_phospho/blob/master/data/raw/41586_2020_2094_MOESM14_ESM.zip |
| PhosPhAt | Prediction data | http://phosphat.uni-hohenheim.de/HiconfPred_psite_20200624.csv | June 24, 2020 | https://github.com/paulati/arabidopsis_phospho/blob/master/data/raw/HiconfPred_psite_20200624.zip |
| MusiteDeep | software | https://www.musite.net/ | 2020 |  |
|  | data from Araport11 protein lists | https://www.arabidopsis.org/download_files/Proteins/Araport11_protein_lists/Araport11_genes.201606.pep.fasta.gz | March 8, 2021 | https://github.com/paulati/arabidopsis_phospho/blob/master/data/raw/Araport11_genes.201606.pep.fasta.gz |
| GO.db version 3.12 |  | <https://bioconductor.org/news/bioc_3_12_release/> | September 10, 2020 |  |
| GOview | Software | http://www.webgestalt.org/2017/GOView/ |  |  |
